# Nitrous Oxyd Again

**Published:** 1867-09

**Authors:** 


### NITROUS OXYD AGAIN.

On the 14th and 16th of the present month we witnessed a complete demonstration of the fact noticed in the first article of this number, viz.: that the patient speedily returns to consciousness while breathing the gas. A patient's knee had been flexed for many years, for the last few to a very acute angle. She took the gas, and the tendons were cut. She took it again and again, that intermittent efforts might be made to straighten the limb, taking it thus four times on the 14th, and three times the 16th. In every instance the anæsthesia was prompt, and the return to consciousness speedy, although the inhalation of atmospheric air was rigidly excluded.

On the 14th, the patient breathed at the rate of twenty inspirations to the minute, till about the twelfth inhalation, and then suddenly dropped to eight or ten to the minute. On the 16th, the change in the character of the breathing took place about the eighth inspiration; and there was a correspondingly earlier return to consciousness. In two or three instances, she breathed nothing for over twenty seconds, yet the countenance and capillary circulation showed no signs of defective oxydation of the blood.

So little constitutional disturbance occurred that the patient, each day, enjoyed a good dinner within a half hour from the close of the operations. We are not giving a report of the operation. That belongs to the surgeon in charge. We are trying to give light on nitrous oxyd. W.

